# Real world clinical outcomes from targeted intraoperative radiotherapy (TARGIT-IORT) during lumpectomy for breast cancer: data from a large cohort at a national cancer institute

**DOI:** 10.3389/fonc.2024.1424630

**Published:** 2024-10-03

**Authors:** Lorenzo Vinante, Jayant Sharad Vaidya, Angela Caroli, Mario Mileto, Erica Piccoli, Michele Avanzo, Loredana Barresi, Marta Marson, Marcella Montico, Lorena Baboci, Tiziana Perin, Martina Urbani, Fabio Puglisi, Maurizio Mascarin, Samuele Massarut

**Affiliations:** ^1^ Radiation Oncology Unit, Centro di Riferimento Oncologico di Aviano (CRO) IRCCS, Aviano, Pordenone, Italy; ^2^ Division of Surgery and Interventional Science, University College London, London, United Kingdom; ^3^ Breast Surgery Unit, Centro di Riferimento Oncologico di Aviano (CRO) IRCCS, Aviano, Pordenone, Italy; ^4^ Medical Phisics Unit, Centro di Riferimento Oncologico di Aviano (CRO) IRCCS, Aviano, Pordenone, Italy; ^5^ Clinical Trial Office, Scientific Direction, Centro di Riferimento Oncologico di Aviano (CRO) IRCCS, Aviano, Pordenone, Italy; ^6^ Immunopathology and Oncologic Biomarkers Unit, Centro di Riferimento Oncologico di Aviano (CRO) IRCCS, Aviano, Pordenone, Italy; ^7^ Pathology Unit, Centro di Riferimento Oncologico di Aviano (CRO) IRCCS, Aviano, Pordenone, Italy; ^8^ Radiology Unit, Centro di Riferimento Oncologico di Aviano (CRO) IRCCS, Aviano, Pordenone, Italy; ^9^ Head, Unit of Medical Oncology and Cancer Prevention, Department of Medical Oncology, Centro di Riferimento Oncologico (CRO), IRCCS, Aviano, Pordenone, Italy; ^10^ Department of Medicine, University of Udine, Udine, Italy

**Keywords:** breast cancer, intraoperative radiotherapy, TARGIT-IORT, real world data, registry

## Abstract

**Introduction:**

Randomised evidence supports the use of partial breast irradiation (PBI) with targeted intraoperative radiotherapy (TARGIT-IORT) for early stage breast cancer, but prospective data from real-world adoption of this technique is also important. The aim of this study was to determine if the outcome reported in TARGIT-A trial could be replicated in large cohort of early stage breast cancer treated with TARGIT-IORT.

**Methods:**

This prospective observational study analysed all patients treated with TARGIT-IORT between 2004 and 2021 in a single national cancer institute. TARGIT-IORT during lumpectomy was performed according to the risk-adapted TARGIT-A protocol using the Intrabeam^®^ device. We analysed the completeness of follow up, 5-year in-breast-tumour-recurrence (IBTR), long term local recurrence free survival, distant disease-free survival, overall survival and breast-cancer-related survival, using the Kaplan-Meier method. A covariate analysis was performed to investigate risk factors for IBTR. We also analysed high grade toxicity events.

**Results:**

The study included 814 patients and the a median follow up was 72 months. The majority of patients (60.3%) received TARGIT-IORT as PBI modality (“exclusive IORT” group); 39.7% received additional EBRT. There was no significant difference between the 5 years IBTR for the whole study population and the “exclusive IORT” cohort (1.6% (95%CI=1.1-2.1%) and 2.5% (95%CI=1.7%-3.3%) respectively). 5 years overall survival and tumour related survival were >95%. In 21% of patients with recurrence, breast was preserved. Radiotherapy toxicity (CTCAE Grade>2) was very rare (0.9%).

**Conclusions:**

This large single institute study found that breast cancer control and survival outcomes with TARGIT-IORT were consistent with TARGIT-A trial results. This “real world” experience confirmed that the randomised evidence showing the value of TARGIT-IORT as partial breast irradiation modality that can be replicated in routine clinical practice.

## Introduction

Partial breast irradiation (PBI) is an effective radiation therapy modality after conservative surgery for early-stage breast cancer. It is based on the finding that the large majority of recurrences after lumpectomy occur close to tumour bed and PBI is planned for irradiation of a small volume of breast tissue surrounding the lumpectomy cavity (1, 2). Several techniques can be used for PBI: LINAC-based external beam radiotherapy (EBRT), brachytherapy, interstitial radiation therapy and intraoperative radiation therapy (IORT).

IORT is an attractive PBI modality because consists in a single radiation therapy application delivered during surgical procedure. PBI with IORT has the advantages of avoiding several daily visits to the hospital to receive standard EBRT and reducing normal tissue toxicity by limiting the irradiation volume to few millimetres around surgical cavity. IORT is usually administered in operating room by portable high energy electrons accelerators or by 50KV X-rays with Intrabeam^®^ system (Carl Zeiss, Oberkochen, Germany).

The efficacy of IORT with Intrabeam^®^ system was investigated in TARGIT-A Trial, an international phase 3, randomised clinical trial that compared PBI with TARGIT-IORT with standard whole breast radiotherapy in early-stage breast cancer patients. In its first publications (3, 4) in-breast tumour recurrence (IBTR) risk of patients treated with TARGIT-IORT immediately after lumpectomy resulted similar to standard EBRT (5-years IBTR=2.1% (IORT) vs. 1.1% (EBRT), p=0.31), whereas delayed (post-pathology) TARGIT-IORT was not non-inferior (5-years IBTR= 5.4 (IORT) vs. 1.7(EBRT), p=0.069).

Updated long-term follow up results (median follow-up=8.6 years) of TARGIT-IORT (5) delivered at time of surgery confirmed the non-inferiority of TARGIT-IORT compared to EBRT (5-years IBRT=2.11% (IORT) vs. 0.95 (EBRT), 90%CI=0.32-1.99) and found no difference in long-term local recurrence-free survival (hazard ratio 1.13, 95% confidence interval 0.91 to 1.41, P=0.28), mastectomy-free survival (0.96, 0.78 to 1.19, P=0.74), distant disease-free survival (0.88, 0.69 to 1.12, P=0.30), overall survival (0.82, 0.63 to 1.05, P=0.13), and breast cancer mortality (1.12, 0.78 to 1.60, P=0.54). Mortality from other causes was significantly reduced (0.59, 0.40 to 0.86, P=0.005) from 9.85% to 5.41%.

TARGIT–IORT is included as a PBI modality in many National and International Guidelines as well as by societies such as the American Cancer Society, European Society for Medical Oncology (ESMO), British Association of Surgical Oncology, UK NICE, and statements such as the St Gallen, Italian, Spanish, Mexican, French, Singaporean consensus/guidelines on treatment of breast cancer (https://targit.org.uk/targit-iort-in-guidelines) albeit in some with the recommendation to maintain a patients’ registry (6, 7) to monitor outcomes in routine clinical practice. For this reason data of outcomes of TARGIT-IORT use in a real-world scenario are needed. In this study we provide new evidence from use of TARGIT-IORT according to TARGIT-A approach in a large cohort of over 800 patients with long and complete follow up. To the best of our knowledge this study represents one of the largest single-institute experience of PBI with TARGIT-IORT delivered immediately after lumpectomy.

## Material and methods

This observational study was performed at Centro di Riferimento Oncologico di Aviano (CRO) IRCCS, Aviano, Italy. TARGIT-IORT treatment was performed between 2004 and 2021 with a breast surgery dedicated Intrabeam^®^ (Carl Zeiss, Oberkochen, Germany) system.

Treatment protocol according TARGIT-A trial was approved by local ethical committee (registration n°CRO 2002.027). Patients enrolled in TARGIT-A (IORT arm) were followed prospectively according, while patients treated outside TARGIT-A trial were collected in a dedicated registry. All patients signed an informed written consent before surgery and IORT and all data were anonymised and collected in a database.

### Patients’ enrolment

All patients included in this analysis were treated according to TARGIT-A trial strategy (8). Patients that received TARGIT-IORT as intentional boost (according to TARGIT Pivotal trial or TARGIT-B trial) or as re-irradiation modality were not eligible for this analysis.

In brief, patients were women with early stage breast cancer, who were candidates for breast conserving surgery. All patients received mammography and breast-axilla ultrasonography. Breast magnetic resonance imaging was not mandatory. Cancer histology was confirmed by needle biopsy. Patients who had their tumours already excised were not suitable for TARGIT-IORT (delayed TARGIT-IORT was not performed). All cases were discussed at multidisciplinary meeting and patients were candidate to TARGIT-IORT in case of unifocal tumour with a diameter ≤3cm, no clinical evidence of positive nodes (cN0), no distant metastases (M0), no lobular histology, no pre-operative systemic therapies.

After surgery all patients were re-discussed at multidisciplinary meeting with final histopatological reports and additional EBRT was recommended based on a combination of factors including the presence of diffuse lymphovascular invasion, lobular histology, extensive *in-situ* component (>25%) and nodes with macro-metastases. All these factors were considered for recommending post-operative whole breast external beam radiotherapy (EBRT).

### Treatment

All patients received conservative surgery and sentinel node biopsy, followed by axillary node dissection in case of presence of macrometastases in sentinel node. Margin status was evaluated by pathologist on fast-frozen tissue sections and macroscopic tissue negative margins before the IORT procedure were required.

TARGIT-IORT was performed immediately after lumpectomy with Intrabeam^®^ system (Carl Zeiss, Oberkochen, Germany), under the same anaesthetic. The energy source was 50kV X-rays and radiation was delivered from the centre of spherical applicators. Applicators diameters ranged between 3 and 5 cm and the beam-on timing was 20-45 minutes. The prescribed dose at 1 cm isodose was 5-6Gy and the dose on applicator surface was 19-21Gy. The distance between applicator surface and skin was measured for all patients and a thickness ≥ 1cm was considered safe to avoid skin necrosis.

If post-operative whole breast EBRT was required the admitted schedules were 50Gy in 25 fractions, 42.56Gy in 16 fractions and 40.05Gy in 15 fractions. 3D-conformal EBRT and intensity modulated radiation therapy techniques were both allowed. No preoperative systemic chemotherapies were permitted. Post-operative endocrine treatments, anti-HER2 drugs or chemotherapy were prescribed according international standard guidelines.

### Follow up

All patients underwent clinical examination at least every six months for the first 5 years after surgery and then annually. Mammogram and breast ultrasonography were performed once a year. Toxicity was evaluated during periodical physical examinations and scored according to Common Terminology Criteria for Adverse Events (CTCAE) scale, version 4.0. Only high grade toxicities (grade ≥3) were recorded. Patients referred to other oncologic centres were periodically interviewed by phone calls to update the follow up status. Patients without any visit or phone contact after surgery were considered as lost at follow up and were excluded from this analysis.

### Statistical analysis

Local and regional recurrences were defined as reappearance of tumour in ipsilateral breast (whether in the tumour bed or elsewhere, and whether a ‘true recurrence’ or a ‘new primary’) and in ipsilateral nodes respectively. The presence of metastases in organs or tissues outside breast or regional nodes was considered as distant recurrences. Time to recurrence was the interval time between surgery and recurrence diagnosis. The date of patients’ last follow-up visit was considered for survival status and length of follow-up. Local control (absence of in breast tumour recurrence, IBTR) was the primary endpoint. Secondary endpoints were risk of regional and distant recurrences, disease-free survival, overall survival, tumour-related survival and incidence of high-grade toxicity.

Local control, regional recurrence-free survival, distant metastases-free survival, disease-free survival, overall survival and tumour related overall survival were estimated using Kaplan Meier methods for the entire cohort and for patients that received exclusive partial breast irradiation with IORT. Covariate analysis for possible risk factors was performed for primary endpoint (local control) both in all study population and in partial breast irradiation cohort. A p-value<0.05% was considered as statistically significant and Bonferonni correction was applied when necessary. For patients treated with exclusive IORT we applied two different PBI consensus guidelines (American Society for Radiation Oncology [ASTRO]2009 (9), Groupe Europeen de Curietherapie and the European Society for Radiotherapy [GEC-ESTRO]2009 (10)] to determine recurrence rate in homogeneous cohorts. Incidence of high grade (CTCAE≥3) toxicity was calculated for all population, exclusive IORT cohort and IORT+EBRT cohort. All statistical analyses were performed with SPSS^®^ software (IBM, US).

## Results

### Patients and treatment

**Figure 1:**
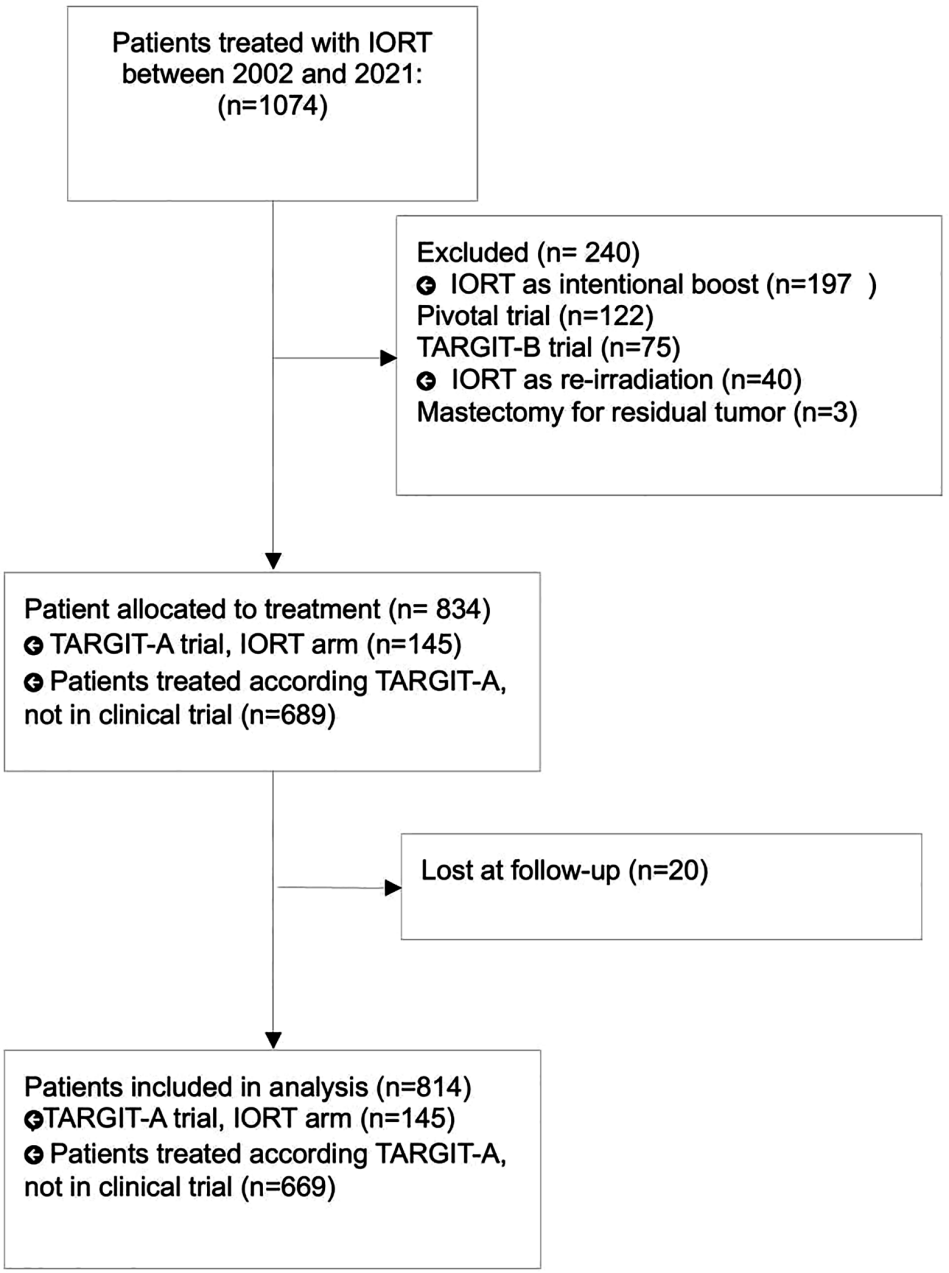
Patients’ selection flowchart.

Between 2002 and 2021, 1074 patients received TARGIT-IORT, consecutively, for early stage breast cancer. 237 patients were excluded because IORT was used as intended boost (Pivotal Trial and TARGIT B Trial) or as reirradiation, 3 patients were excluded because received mastectomy for tumor residual immediately after lumpectomy and 20 patients were considered lost at follow up. Finally, 814 patients were eligible for analysis. Patients’ selection flowchart was described in **Figure 1**.

All patients met the inclusion criteria for TARGIT-A trial (8). Patients’ and tumour characteristics were summarised in **Table 1**. Median lumpectomy specimen volume was 63 grams (range: 11-495 grams). TARGIT-IORT dose prescription was 5Gy to 1cm isodose (until 2012, 37.4% of cases) and 6Gy to 1cm isodose (from 2013, 62.6% of cases). TARGIT-IORT was considered as PBI modality (“exclusive IORT” cohort) in 491 (60.3%) patients or as unintended tumour bed boost followed by EBRT (“IORT+EBRT” cohort) in 323 (39.7%) cases.

**Table 1:** Patients’ and tumour characteristics and treatments.

Characteristics	N° patients (%)
Age (years)	
<50 50-59 60-69 ≥70	58 (7.1) 201 (24.7) 285 (35) 270 (33.2)
Tumour side	
Right Left	382 (46.9) 432 (53.1)
Histology	
NST carcinoma Lobular carcinoma NST + lobular carcinoma Intracystic carcinoma	757 (93) 52 (6.4) 3 (0.4) 2 (0.2)
T stage	
pTis pT1a pT1b pT1c pT2	2 (0.2) 75 (9.2) 396 (48.6) 307 (37.7) 33 (4.1)
N stage	
pN0 pN1mic pN1a pN2a pN3a Unknown	594 (73) 92 (11.3) 100 (12.3) 5 (0.6) 4 (0.5) 2 (0.2)
Grade	
1 2 3 Unknown	96 (11.8) 487 (59.8) 226 (27.8) 5 (0.6)
Diffuse lymphovascular invasion	
Absent Present Unknown	573 (70.4) 237 (29.1) 4 (0.5)
Ductal carcinoma *in situ* component >25%	
Absent Present Unknown	758 (93.1) 53 (6.5) 3 (0.4)
Molecular histotype	
Luminal (A or B) HER2 enriched Triple negative Unknown	717 (88.1) 46 (5.7) 45 (5.5) 6 (0.7)
Regional node surgery	
Sentinel node(s) biopsy Axillary node dissection Not performed	686 (84.3) 126 (15.5) 2 (0.2)
TARGIT-IORT dose	
5Gy at 1cm isodose 6Gy at 1cm isodose	307 (37.7) 507 (62.3)
TARGIT-IORT applicator diameter (mm)	
30 35 40 45 50 Unknown	4 (0.5) 57 (7.0) 157 (19.3) 211 (25.9) 378 (46.4) 7 (0.8)
EBRT after TARGIT-IORT	
Yes No	323 (39.7) 491 (60.3)
EBRT volume	
Whole breast Whole breast and regional nodes Not applicable	313 (38.5) 9 (1.1) 492 (60.4)
EBRT dose	
50Gy in 25 fractions 42.56Gy in 16 fractions 40.05Gy in 15 fractions 26Gy in 5 fractions Not applicable	98 (12) 35 (4.3) 185 (22.7) 2 (0.2) 494 (60.7)
Chemotherapy	
Yes No Unknown	137 (16.8) 666 (81.8) 11 (1.4)
Endocrine therapy	
Yes No Unknown	654 (80.3) 153 (18.8) 7 (0.9)
Anti HER2 therapy	
Yes No Unknown	46 (5.7) 762 (93.6) 6 (0.7)

Patients that received IORT+EBRT had one or more of the following features: positive nodes (n=163, 51%), diffuse lymphovascular invasion (n=140, 43%), lobular histology (n=43, 13%) or ductal carcinoma *in situ* component ≥25% (n=25, 8%). When indicated, EBRT was administered with standard fractionated (32%) or hypofractionated (68%) regimen.

A significant proportion of patients had grade 3 tumours (28% of whole population and 25% exclusive IORT” cohort), diffuse lymphovascular invasion (29% of the whole population and 20% of “exclusive IORT” cohort) and positive nodes (27% of the whole population and 10% of “exclusive IORT” cohort). Additional details regarding surgery, radiation therapy or systemic therapies are reported in **Table 1**.

### Outcome measures

The completeness of follow up was 83% at 5 years (for patients treated within 2018) and median follow up was 72 months (range: 0.3-18.3 years). Overall 42/814 (0.86 per 100 person-years) patients had a local recurrence, 29/491 (0.98 per 100 person-years) in “exclusive IORT” cohort and 13/323 (0.67 per 100 person-years) in “IORT+EBRT” group. A total of 62 deaths were reported, four fifths of the total deaths, (n=50) were not due to breast cancer while in one-fifth (n=12) tumor progression was considered the cause of death.

Five-years estimated IBTR risk and survival without IBTR (event=local recurrence or death) were 1.6% (95%CI=1.1-2.1%) and 95.2% (95%CI=94.4%-96%), respectively. Five-years overall and breast cancer specific survival rates were 96.6% (95%CI=95.9%-97.3%) and 99% (95%CI=98.6%-99.4%), respectively.

**Figure 2:**
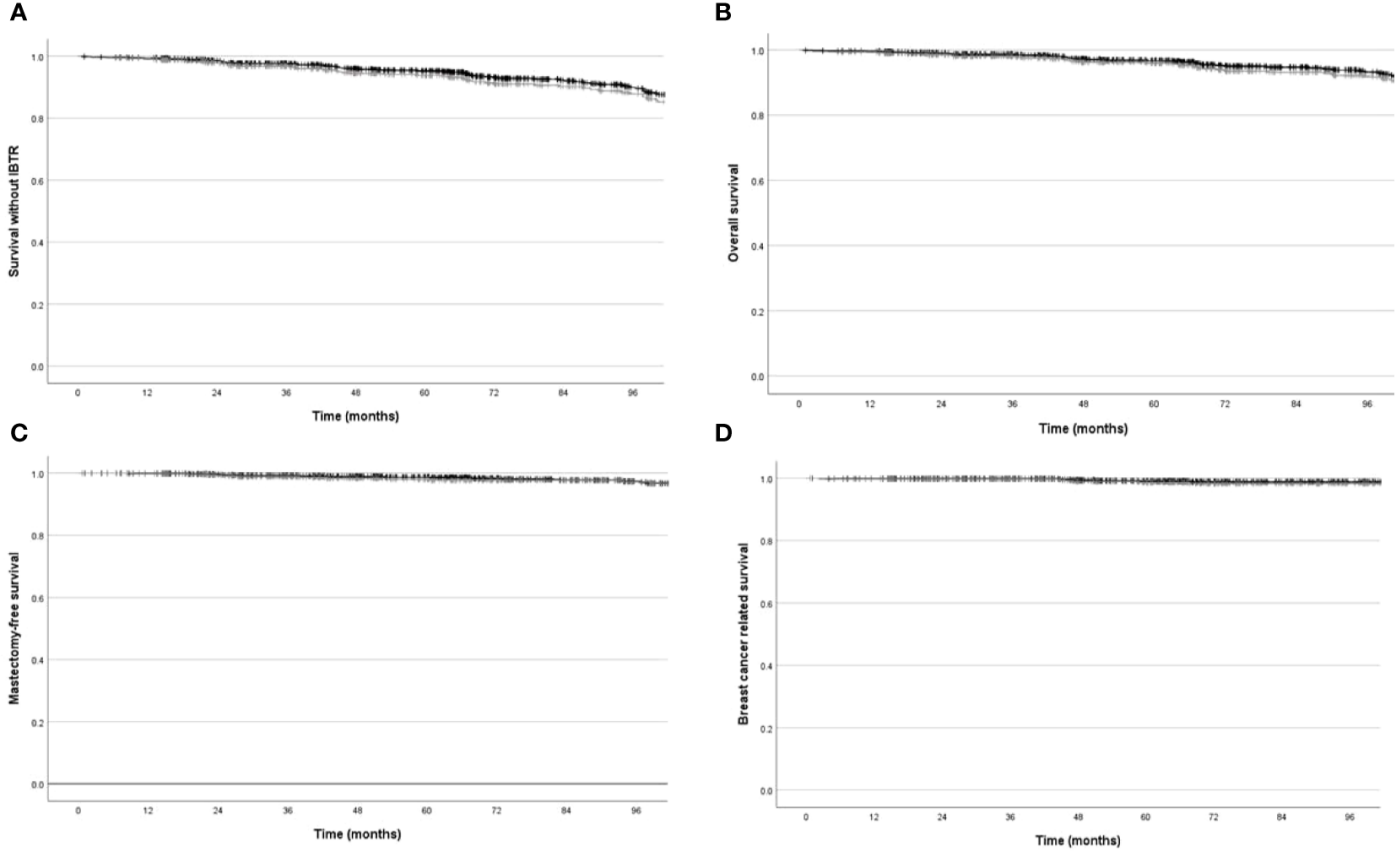
Local recurrence free survival, mastectomy-free survival, overall survival, breast cancer related survival, estimated with Kaplan Meier methods in overall cohort (black line) and in “exclusive IORT” cohort (gray line). **(A)** Local recurrence free survival (p=NS). **(B)** Mastectomy-free survival (p=NS). **(C)** Overall survival (p=NS). **(D)** Breast cancer related survival (p=NS).

Similar results were obtained in “exclusive IORT” cohort: Five-years estimated IBTR risk and survival without IBRT (event=local recurrence or death) were 2.5% (95%CI=1.7%-3.3%) and 93.8% (95%CI=92.6%-95%), respectively. Five-years overall and tumour-related survival were 95.9% (95%CI=94.9%-96.9%) and 98.6% (95%CI=98%-99.2%), respectively. Kaplan Meier plots are shown in **Figure 2**. Five-years Kaplan Meier estimates of outcome measure were summarised in **Table 2**.

**Table 2:** Five-years Kaplan Meier estimates of outcomes measures for all population and for exclusive IORT cohort.

Outcomes	All study population	Exclusive IORT cohort
	Kaplan-Meier estimates (95%CI)	Kaplan-Meier estimates (95%CI)
5 years local recurrence-free survival	95.2% (94.4%-96%)	93.8% (92.6%-95%)
5 years mastectomy free survival	98.7% (98.3%-99.1%)	98.1% (97.4%-98.8%)
5 years regional recurrence-free survival	99% (98.6%-99.4%)	98.5% (97.9%-99.1%)
5 years distant recurrence-free survival	98.4% (97.9%-98.9%)	98.2% (97.5%-98.9%)
5 years overall survival	96.6% (95.9%-97.3%)	95.9% (94.9%-96.9%)
5 years tumour related overall survival	99% (98.6%-99.4%)	98.6% (98%-99.2%)
5 years not tumour related overall survival	97.4% (96.8%-98%)	97.2% (96.4%-98%)

### In-breast recurrence analysis

A total of 42 in-breast tumour recurrences were registered during follow up, 29 in “exclusive IORT” cohort and 13 in “IORT+EBRT” cohort. Median time between surgery and local recurrence was more than 8 years (101 months, range: 9-161 moths). Tumour histology at recurrence was infiltrative carcinoma in 40 (95%) cases and ductal *in situ* carcinoma in 2 (5%). The tumor recurrence site was reported in 34 patients and was inside the previous tumor bed (“in-field” recurrence) or in another breast quadrant in 9 (26%) and 25 (74%) of cases, respectively.

A second surgery was performed in 39 (93%) patients, while 3 (7%) cases with skin infiltration received only a punch biopsy. Second surgery consisted in mastectomy in 30 cases (71%) and a second lumpectomy in 9 cases (21%). 5-years mastectomy free survival in all study population and in exclusive IORT cohort was 98.7% (98.3%-99.1%) and 98.1% (97.4%-98.8%), respectively. Patients treated with a second lumpectomy received post-operative EBRT. Three of 42(7%) patients experienced a second local recurrence. Seven out of 42(17%) of patients with local recurrence died (due to tumour in 4 patients or to other causes in 3 patients).

Age<60 years, T2 stage, positive nodes, lobular histology, grading G3, diffuse lymph-vascular invasion, ductal carcinoma *in situ >*25%, absence of estrogenic receptors and HER-2 over-expression were considered as possible risk factors for IBRT, but none of these factors were statistically related (p=NS) with IBTR, both in all population and in exclusive IORT cohort (**Table 3**). Local recurrence rates were also calculated in two different cohorts defined according ASTRO 2009 (9) and GEC-ESTRO 2009 (10) consensus guidelines for exclusive PBI (**Table 4**). ASTRO 2009 and GEC-ESTRO 2009 criteria were satisfied in 205/49 (42%) and 306/491 (62%) patients, respectively. 5-years IBTR rate according to ASTRO 2009 and GEC-ESTRO 2009 was 2.0% (95%CI=0.8%-3.2%) and 1.8% (95%CI=0.9%-2.7%), respectively.

**Table 3:** Covariate analysis for local recurrence risk factors.

Risk factors	All population			Exclusive IORT		
	IBTR No	IBTR Yes	p-value	IBTR No	IBTR Yes	p-value
Age						
<60 years ≥60 years	244 528	15 27	NS	130 332	10 19	NS
T stage						
T1, pTis T2	742 29	38 4	NS	448 13	28 1	NS
N stage						
0 N+	565 199	29 12	NS	419 43	24 5	NS
Histology						
Not ILC ILC only	723 49	39 3	NS	\	\	\
Lymph-vascular invasion						
Absent Present	545 223	28 14	NS	373 87	19 10	NS
DCIS>25%						
Absent Present	720 49	38 4	NS	434 26	27 2	NS
Grading						
G1-G2 G3	554 213	29 13	NS	347 111	18 11	NS
ER receptor status						
Positive Negative	715 51	39 3	NS	424 33	26 3	NS
HER-2 over-expression						
Yes No	723 44	40 2	NS	438 20	27 2	NS

**Table 4:** Partial breast irradiation guidelines applied to exclusive IORT group.

Parameter	GEC-ESTRO (2009)	ASTRO (2009)	Present study
Age	≥50	>60	≥50
Tumor diameter	≤3	≤2	≤3
Tumor grading	Any	Any	Any
Tumor histology	IDC only	ICD only	ICD, mixed ICD + ILC
Ductal *in situ* carcinoma only	No	No	Only intracystic papillary carcinoma
Node status	pN0	pN0	pN0 or pN1mic
Margins	≥2mm	≥2mm	Negative
Lymphovascular invasion	Absent	Absent	Absent or focal
Estrogen receptor	Any	Positive	Any
HER2 over-expression	No	No	Yes
N° patients	306	205	491
5-years IBTR risk %(95%CI)	1.8 (0.9-2.7)	2.0 (0.8-3.2)	2.5% (1.7-3.3)

### Toxicity

In all study population high grade side effects occurred in 7/814 patients (0.9%): 1 skin necrosis, 3 Grade-3 fibrosis and 3 radiation induced angiosarcomas. Incidence of Grade 3-4 toxicity was 0.4% in patients treated with exclusive IORT and 1.5% after IORT plus EBRT. The cases of radiation induced angiosarcoma were observed 11 years after exclusive IORT in one case and 5 years after IORT+EBRT in 2 cases. All cases were treated with mastectomy alone.

## Discussion

Several clinical phase-3 trials (11–14) have demonstrated the non-inferiority of PBI in comparison to whole breast EBRT in selected early stage breast cancer patients. Different radiation therapy techniques e.g. EBRT, interstitial radiation therapy, brachytherapy and IORT could be used for PBI.

Our study provides new “real world” data of PBI delivered with TARGIT-IORT. After a median follow up of 72 months, 5-year IBTR risk was no different in our study population treated with the risk-adapted approach (1.6%) compared with those who had exclusive TARGIT-IORT, with an overall survival >95% in both series. High-grade toxicity events were very rare, with an incidence of 0.9%.

Our long term data were consistent with TARGIT-A trial results, that reported a 5-year IBTR of 2.11% in the IORT arm (5). Therefore our findings confirm the favourable results of TARGIT-IORT when applied immediately after lumpectomy.

Other “real world” data of TARGIT-IORT as PBI modality were reported in many registry studies from Europe (including France, Denmark, Spain, Germany, etc.), Israel, USA, Canada, South America and others (15–19). Tellet et al. (15) reported a 5-years IBRT risk of 1.7% for patients treated according TARGIT-A and 1.5% in IORT alone group in low-risk luminal breast cancers, while we applied less restrictive inclusion criteria, similar to TARGIT-A trial. In our population of patients treated with exclusive IORT, the 5-years IBTR would be 2.0% and 1.8% according to ASTRO 2009 (9) and GEC-ESTRO 2009 (10) criteria, respectively. Survival outcomes (5-years overall survival=96.5% and tumour-specific overall survival=98.9%) and toxicity profile (Grade3-4 toxicity=0.6%) reported by Tellet et al. (15) were consistent with our data. Many other worldwide experiences (16–18) confirmed these favourable clinical outcomes. There are currently over 250 published papers that support the use of TARGIT-IORT for breast cancer (see https://bit.ly/TARGIT-IORT-Bibliography for a live database). Only one of these 250 studies (19) has described a higher risk of local recurrence with TARGIT-IORT, but this study presented several limitations (e.g. poor patient selection, highly incomplete and selective follow up information (e.g., >50% loss to follow up), and non-compliance with the TARGIT-A protocol). Our large single institute experience of over 800 patients is of a homogeneous study population. Treated with a specialised team, and has a near-complete and robust prospective follow up. We found that TARGIT-IORT has excellent early and long-term local control and survival outcomes for breast cancer patients.

A meta-analysis (20) based on 4918 patients treated with EBRT or brachytherapy in 7 randomised clinical trials revealed a 5years IBTR of 1.8% (95% HPD 0.68-3.2). By PBI technique, the 5-year rate of IBTR rate for EBRT was 1.7% and 2.2% for brachytherapy. In GEC-ESTRO brachytherapy trial (12) the 5-year cumulative incidence of local recurrence was 1.44% (95% CI 0.51-2.38), an incidence similar to our study in patient cohort selected with the same GEC-ESTRO criteria (1.8%, 95%CI=0.9%-2.7%). Conversely, ELIOT trial (21), a phase 3 randomised trial of PBI with electrons energy IORT, reported a 5-years IBTR = 4.2% in IORT arm. IORT and other PBI techniques have not been compared head-to-head in a clinical trial and inclusion criteria were often different within PBI studies. For this reason, a direct comparison between our data and series treated with other PBI modality was not easy. However, our data favourably compared with ELIOT results and were similar to EBRT and brachytherapy series. Similar findings were reported by Mi et al. (22) in a propensity score matching study with a 5 year local control of 2.3% in TARGIT-IORT group and 1.6% in EBRT group (p=0.66). Also, this group found that TARGIT-IORT had an improved overall survival compared to no-radiotherapy in a propensity match study of a large SEER dataset including over 1600 patients who had received IORT in the USA (23).

We investigated possible risk factors as age<60years, tumour size, node status, histology, grading, oestrogen receptor and HER2 status, lymphovascular invasion and diffuse ductal carcinoma *in situ* presence. We found that these factors did not correlate with an increase of IBTR, potentially because of the overall low risk of IBTR. These results were consistent with a secondary analysis of TARGIT-A trial (12), in which these factors did not influence a difference in local control between TARGIT-IORT and EBRT.

Additionally, Vaidya et al. (24) reported a favourable prognostic significance of local recurrence after TARGIT-IORT in comparison to EBRT was reported, with a lower risk of distant metastases and tumour related deaths when the recurrence occurred after TARGIT-IORT. We detected that the majority (74%) of local recurrences were outside the tumor bed, reflecting the efficacy of TARGIT-IORT to prevent recurrences inside the high radiation dose area. Additionally, in patients that experienced local recurrence we detected only 3 cases (7.1%) of distant metastases and 4 cases (9.5%) of tumour related deaths. Our data were in line with the TARGIT-A trial that also reported a low (9%) hazard of death following local recurrence (24).

It is important to recognise that results amongst those who received exclusive IORT the local recurrence rates were very low and were substantially better than those reported for much older and lower risk women in whom no radiotherapy is given, (e.g., 2.5% vs 4.1% in the PRIME-II trial (25).

From our data we confirmed good tolerance of TARGIT-IORT procedure with a very low incidence rate of Grade 3-4 toxicity (0.9%), confirming the high-level of safety of TARGIT-IORT procedure. It is of interest that in this paper we describe the first case of radiation induced angiosarcoma in patients treated with TARGIT-IORT.

### Strength and limitation

Strengths: The longest follow up time is 17 years and the assessment of the follow up completeness was adequate (83%) for the first 5 years; therefore the data were reliable for statistical analysis. We have data on actual treatment received, toxicity and local recurrence in almost all patients.

Limitations: we had prospectively recorded only Grade 3-4 toxicity, but this is the case even in prospective randomised trials. Secondly, EBRT was used after IORT in a larger proportion (39.7%) of cases compared to the 20-22% in the TARGIT-A trial. The reason was because we were being very cautious when treating patients outside a clinical trial, and EBRT was added to many of these patients was because of just micrometastasis in the lymph node. We recommend using the web tool derived from long-term results of the TARGIT-A trial (24) (see https://targit.org.uk/addrt) can guide decisions about adding EBRT after TARGIT-IORT.

## Conclusions

This large, single-institutional study, confirmed with “real world” data the results of TARGIT-A trial (5). In particular, we confirmed a low risk of local recurrence in patients treated with TARGIT. In our single institute study homogeneous selection criteria and treatments were performed. We strongly believe that the most critical part of the entire procedure was proper execution of the risk-adapted approach, meticulous carrying out of the TARGIT-IORT procedure, and following the TARGIT-A protocol in preparing our institutional protocol (26).

In conclusion, our data strongly supports the use of TARGIT-IORT as a PBI technique in selected early stage breast cancer patients as per the inclusion criteria of the TARGIT-A trial. The treatment was safe and effective and found a low risk of local recurrences. These results were comparable with those of long term follow up results of the randomised TARGIT-A trial. Moreover, our findings, showed that outcomes in randomised clinical trials can be replicated in the real-world situation.

## Data Availability

The raw data supporting the conclusions of this article will be made available by the authors, without undue reservation.
